# National survey on management practices for adhesional small bowel obstruction and nasogastric tube use among general surgeons in Australia

**DOI:** 10.1186/s12893-026-03587-8

**Published:** 2026-06-20

**Authors:** Ali Al-Mashat, Anaan Fareed, Stephen Smith, Johnathan Gani

**Affiliations:** 1 Surgical Services, John Hunter Hospital, Newcastle, NSW Australia; 2 School of Medicine and Public Health, University of Newcastle, Newcastle, NSW Australia; 3 Hunter Medical Research Institute, Newcastle, NSW Australia; 4 Hunter Surgical Clinical Research Unit, Newcastle, NSW Australia; 5 Department of Surgery, Calvary Mater Newcastle, 20 Edith St. Waratah, Newcastle, NSW 2298 Australia

**Keywords:** Small bowel obstruction, Adhesions, Nasogastric tube, Surgery, Surveys and questionnaires

## Abstract

**Background:**

The nasogastric tube (NGT) is recommended routinely by current guidelines in the non-operative management of adhesional small bowel obstruction (ASBO). However, several retrospective studies have reported poorer outcomes in ASBO patients managed with an NGT. We sought to examine current practices among Australian General Surgeons in managing ASBO and using NGTs, assess the presence of surgical equipoise for a potential randomised controlled trial (RCT), and evaluate interest in multi-centre collaboration.

**Methods:**

In October 2021, we conducted an online survey of all active members of General Surgeons Australia using a REDCap^®^ questionnaire distributed via email. The survey included clinical scenarios of small bowel obstruction and questions on demographics, management practices, and clinical reasoning. Descriptive statistics were used to summarise all relevant variables.

**Results:**

Of the 893 emails sent, 181 responses were received (20%). Most respondents were from New South Wales (47%) and had subspecialty training (71%). An NGT was used in over 90% of ASBO cases by 42% of respondents. 64% believed NGT use could prevent surgery, and 93% viewed it as having a therapeutic role. Vomiting was the most common indication for insertion, and 69% believed it reduces aspiration risk. Gastrografin^®^ was used by 96% of surgeons. 42% expressed willingness to participate in an RCT evaluating NGT use.

**Conclusions:**

There is considerable variation among surgeons in their beliefs regarding its utility. The observed variation in practice patterns and underlying beliefs reflects ongoing clinical uncertainty. Given the exploratory nature of the survey and its response rate, these findings should be interpreted as reflecting practice variability, rather than establishing consensus, and may inform the design of further investigation through a RCT.

**Supplementary Information:**

The online version contains supplementary material available at https://doi.org/10.1186/s12893-026-03587-8.

## Background

Adhesional small bowel obstruction (ASBO) is a common cause of hospital admissions, associated with significant morbidity and mortality [1]. Unlike other forms of small bowel obstruction (SBO), ASBO is the most common and is frequently managed non-operatively [2, 3]. Traditionally, nasogastric tubes (NGTs) have been integral to the management of ASBO, as outlined in the Bologna guidelines from the World Journal of Emergency Surgery [4]. However, unlike other aspects of ASBO management, such as the Gastrograffin^®^ challenge, which is supported by level one evidence [5], the routine use of NGTs in ASBO remains a topic of debate. This is largely due to the limited and low-quality evidence supporting its efficacy [6–8], as well as concerns regarding the potential discomfort and complications NGTs may cause patients [9]. 

The absence of standardised protocolised care for ASBO has led to significant variations and inconsistencies in clinical practice. Combined with the lack of robust evidence supporting NGT use, this highlights the need for a better understanding of how surgeons approach the management of ASBO. We conducted a survey to capture the current practices of Australian General Surgeons regarding ASBO management and their use of NGT decompression. Our objectives were to determine whether surgical equipoise exists for a randomised controlled trial (RCT) comparing NGT use against non-use in ASBO and gauge interest in a multicentre study.

## Methods

A questionnaire survey (Appendix S1) was designed and distributed to all active members of General Surgeons Australia (GSA) via email through an attached web link [10]. GSA is a not-for profit governing body of practicing General Surgeons in Australia with membership granted to surgeons registered with the Royal Australian College of Surgeons (RACS).

The survey was designed through a panel discussion of 9 general surgeons from the Acute General Surgical Unit at John Hunter Hospital. Questions were formulated to explore current management practices and beliefs surrounding ASBO and the use of NGTs. The draft questionnaire was subsequently piloted among general surgeons across the Hunter New England Area Health Service before final distribution.

The survey was administered, and data were stored using the Research Electronic Data Capture (REDCap^®^) database. The study was conducted between October 2021 and March 2022. The questionnaire collected demographical data for each respondent, including level of experience, fellowship and subspecialty training, location and involvement in acute surgical on call practice. The survey link was distributed by GSA via email to all financial members who were fellows and consultants, with two reminder emails sent to non-responders. Participation was voluntary, no incentives were offered, and the survey was not externally advertised. Although, it was informally discussed at the RACS Annual Scientific Congress (ASC) during an initial presentation. Respondents were presented with three clinical scenarios and questions designed to explore their self-reported management practices and beliefs in the care of ASBO and the use of NGTs. Multiple-choice questions were used, with some allowing for free text responses. The survey questionnaire did not seek identifiable information from the respondent except where the option for an expression of interest in a multicentre study was selected, in which their name and contact email was obtained. Descriptive statistics were reported for all relevant variables, including frequencies and percentages for categorical variables. Analysis was performed and figures were generated using Microsoft Excel. This research was approved by the Hunter New England Human Research Ethics Committee of the Hunter New England Local Health District (reference: 2021//ETH00724) with subsequent approval from GSA.

## Results

Survey responses (including partially completed surveys) were received from 181 surgeons out of 893 emails sent (20% response rate). Demographical data is outlined in Table 1. The respondents were predominantly from metropolitan or regional hospitals (94%), practicing in the state of New South Wales (47%). Most of the surgeons identified as completing fellowship or subspecialty training (71%), with colorectal surgery being the most common subspecialty (26%). Respondents mainly reported having 1–5 years of specialist experience (39%). Almost all were engaged in acute surgical on-call duties at the time of the survey (94%), with approximately half managing 4 or more cases of ASBO per month (52%).

**Table 1:** Demographic data of survey respondents

Characteristic	N (%)
Practice location (n=179)	
Metropolitan	103 (58)
Regional	65 (36)
Rural	11 (6)
State (n=174)	
New South Wales	81 (47)
Queensland	32 (18)
Victoria	39 (22)
Western Australia	9 (5)
South Australia	12 (7)
Tasmania	1 (1)
Specialist experience (years), (n=179)	
1-5	70 (39)
6-10	23 (13)
11-15	22 (12)
>15	64 (36)
Surgical subspecialty training (n= 180)	
Yes	128 (71)
No	52 (29)
Type of sub-speciality, (n=180)*	
Breast	29 (16)
Endocrine	22 (12)
GIT	27 (15)
Colorectal	46 (26)
Trauma	13 (7)
Rural Generalist	8 (4)
Head and neck	4 (2)
Surgical oncology	23 (13)
Transplant	4 (2)
Vascular	1 (1)
HPB	11 (6)
Bariatric surgery	10 (6)
Other	
Upper GI/Oesophogastric	3 (2)
ASU/Emergency Surgery	1 (1)
Unspecified	1 (1)
Worked in acute/general surgical on call (n=179)	
Yes	169 (94)
No	10 (6)
Number of ASBO patients treated per month (n=169)	
0-3	81 (48)
4-5	70 (41)
6-10	14 (8)
>10	4 (2)

^*^Respondents were able to select multiple responses

Forty-two percent of respondents would advise the use of a NGT in more than 90% of ASBO cases. More than half of the respondents believed that a NGT can reduce the need for surgery in patients with ASBO (64%). Out of 133 surgeons who responded to whether an NGT could be therapeutic, 124 believed yes (93%). Of those, 121 selected that an NGT was therapeutic because it facilitated resolution (98%) and 44 selected that an NGT was therapeutic because it prevented surgery (35%); respondents could select both options. In ASBO requiring surgical intervention, 80% of respondents said they would insert an NGT pre-operatively. Most respondents believed that optimal NGT use reduces aspiration risk (69%), while some thought it increases it (9%) or did not change it (14%). Free text responses to aspiration risk were provided by some (8%), including that NGT reduces aspiration triggered by vomiting but may increase the risk of reflux-induced aspiration. Other responses included a lower risk in ASBO patients with persistent vomiting but a higher risk in those undergoing general anaesthesia or that are comatose. Some respondents stated that the risk varies among patients, stating it could reduce aspiration severity and volume while possibly increasing the risk of low-volume aspiration.

**Fig. 1:**
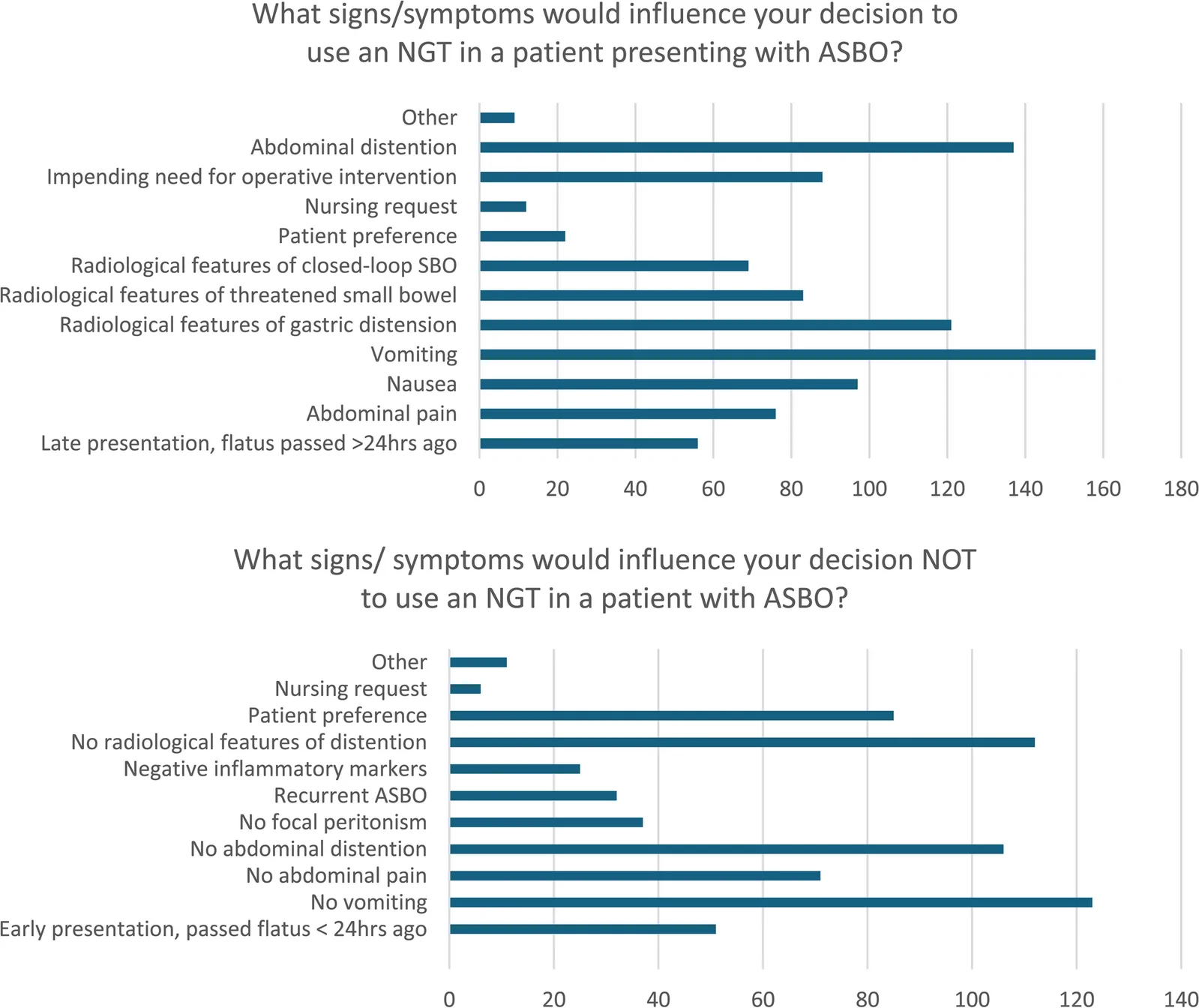
Bar graphs using absolute numbers, illustrating surgeon responses to indications for and against NGT use^*^

For maintenance of an NGT, free drainage was preferred amongst surgeons (63%). Gastrograffin^®^ was widely used in ASBO (96%), with 47% choosing either oral or NGT for administration. The optimal time frame for non-operative management was deemed to be 48–72 h. However, 83% of respondents were willing to manage a patient with ASBO conservatively using a NGT for this timeframe, compared to only 68% without an NGT. 6% of respondents would not manage an ASBO patient non-operatively without an NGT. Fig. 1 illustrates factors influencing surgeons’ decisions for and against NGT insertion and Table 2 outlines the free text responses. When presented with a clinical scenario depicting a vomiting ASBO patient, 97% opted for NGT insertion compared to only 33% when vomiting was absent in the scenario. When the scenario depicted a closed loop bowel obstruction, the majority indicated they would insert an NGT (91%). Almost half of the respondents (42%) expressed willingness to participate in an RCT investigating NGT use. Survey response numbers and percentages are outlined in Table 3.

**Table 2:** Free text responses for reasons to insert or not insert an NGT

Free text responses for reasons to insert an NGT	Free text responses for reasons not to insert an NGT
- Elderly patients or patients with dementia. - Prior to Gastrograffin^®^ administration to ensure emptying of gastric contents. - Routinely for ASBO/SBO. - Profuse vomiting with a proximal obstruction. - Significantly distended stomach on CT. - Radiological features of an ASBO with upstream dilatation and gastric distention.	- Patient refusal. - Unsuccessful insertion. - Patient improving, passing flatus or a younger age group. - Patients with recurrent ASBO. - Threatened bowel, closed loop obstruction, or ASBO with impending surgery. - Diagnosis of ASBO is uncertain. - Early return to gut function e.g. passing flatus, between time of the scan and surgical review. - Patient unable to tolerate. - Palliation.

**Table 3:** Management practices and beliefs with NGT use in ASBO

Survey question	N (%)
Can NGT insertion save some patients with ASBO from operative intervention? (n=168)	
Yes	107 (64)
No	32 (19)
Undecided/unsure	29 (17)
Can NGT insertion be therapeutic for ASBO? (n=133)	
Yes	124 (93)
No	9 (7)
Inserting an NGT is therapeutic because of? (n=124)*	
Preventing need for surgery	44 (35)
Assisting in the resolution of ASBO	121 (98)
How frequently would you advise the use of a NGT for patients with ASBO? (n=169)	
Never	0 (0)
0-10%	5 (3)
11-30%	11 (7)
31-70%	33 (20)
71-90%	49 (29)
>90%	71 (42)
Do you think the optimal use of an NGT changes the risk of aspiration with ASBO? (n=168)	
Increases the risk	15 (9)
No change	24 (14)
Decreases the risk	116 (69)
Other	13 (8)
What is your preference for the maintenance of an NGT in ASBO (n=167)	
Free drainage	106 (63)
Manual aspiration q4hrly	47 (28)
Low continuous suction	14 (8)
Do you use Gastrograffin® in ASBO management? (n=169)	
Yes	163 (96)
No	6 (4)
How do you administer Gastrograffin®? (n=163)	
Orally	13 (8)
NGT	74 (45)
Either	76 (47)
Do you insert an NGT before an emergency operation for a patient with ASBO? (n=168)	
Yes	135 (80)
No	14 (8)
Only if requested by an anaesthetist	16 (10)
Undecided/unsure	3 (2)
Would you consider participating in a randomised controlled trial (n=165)	
Yes	69 (42)
No	96 (58)

^*^Respondents were able to select multiple responses

## Discussion

Our study represents the first national survey of Australian General Surgeons that examines current management practices for ASBO and the use of NGTs. Our findings indicate a general adherence to established ASBO management guidelines [4]. The majority of surgeons reported frequent use of NGT decompression, and nearly all reported using Gastrograffin^®^ in their practice. Rather than demonstrating consensus, the findings show substantial variation in practice patterns and beliefs, particularly regarding indications for NGT insertion, perceived therapeutic benefit, and its role in preventing operative intervention.

Despite guidelines advocating for universal NGT use in ASBO [4, 11, 12], our findings suggest that Australian surgeons are taking a more selective approach. Only 42% reported routine use of NGTs (i.e. in > 90% of ASBO cases). Decisions to insert an NGT were primarily based on clinical indicators such as nausea and vomiting, abdominal distension, suspected closed loop obstruction, or the need for surgery. However, there are currently no protocols guiding patient selection for NGT use. Interestingly, vomiting was the most common reason for NGT insertion, whereas the absence of vomiting and lack of radiological gastric distention were the most common reasons for not inserting. Yet, our review of the literature does not support this practice. One study, which stratified patients by gastric volume to assess whether NGTs reduced vomiting in those with larger volumes, found no significant association [8]. Another study by Fonseca et al. reported higher rates of pneumonia in patients managed with an NGT [6]. 

Given the lack of RCTs and the limited number of retrospective studies available, it appears that the selective use of NGTs reflects individual surgeon judgment and beliefs about their role in ASBO management. This may explain why retrospective comparisons often show poorer outcomes in the NGT group [6–8], potentially because they are preferred for use in patients with greater disease severity. Our survey showed that surgeons preferred NGT use in patients needing operative intervention. This raises the question: if a patient appears unwell enough to require an NGT, should earlier operative management be considered instead? These findings suggest that the role of the NGT is poorly understood, with conflicting perspectives among surgeons regarding its clinical value and ability to prevent surgery. Such variation in beliefs, particularly in the absence of clear evidence, reinforces the uncertainty around whether the NGT itself influences outcomes. A RCT is therefore essential to define the true role of NGTs in ASBO and to guide future clinical practice.

## Limitations

The primary limitation of our study is the lower than anticipated response rate and the number of partially completed surveys returned, posing the threat of underrepresentation and respondent bias. While the length of the survey may have contributed to this, it is also important to consider the impact of longstanding surgical dogma, particularly regarding interventions like the NGT, which has been central to management of bowel obstructions for over a century. Furthermore, as with most survey-based studies, the reliance on predominantly structured, fixed-response questions may have constrained the ability to capture the full complexity of clinical reasoning and perspectives behind management choices. Nevertheless, despite the low response rate, which includes a sample population that predominantly believes in the therapeutic role of the NGT, our survey indicates that a discernible proportion of surgeons are still willing to participate in a clinical trial assessing its use.

## Conclusions

There is considerable variation among surgeons in their beliefs regarding its utility, with nearly half of the survey respondents indicating a willingness to participate in a prospective clinical trial. The observed variation in practice patterns and underlying beliefs reflects ongoing clinical uncertainty. Given the exploratory nature of the survey and its response rate, these findings should be interpreted as reflecting practice variability, rather than establishing consensus, and may inform the design of further investigation through a RCT.

## Supplementary Information


Supplementary Material 1.


## Data Availability

The datasets generated and analysed during the current study are available from the corresponding author on reasonable request.

## Abbreviations

NGT: Nasogastric tube
RCT: Randomised controlled trial
ASBO: Adhesional small bowel obstruction
GSA: General Surgeons Australia
REDCap®: Research Electronic Data Capture
