# Supplementary material for: National survey on management practices for adhesional small bowel obstruction and nasogastric tube use among general surgeons in Australia
Source: BMC Surg. 2026 Jun 20;26:591. doi: 10.1186/s12893-026-03587-8 (PMC13536556; doi:10.1186/s12893-026-03587-8)
Supplement: Supplementary file 1 [file 12893_2026_3587_MOESM1_ESM.pdf]

A survey of nasogastric tube use for adhesional small bowel obstruction

Dear Colleague,

This survey aims to explore current practise with respect to nasogastric tube usage in the management of adhesional small bowel obstruction (ASBO). On average, this survey will take less than 10 minutes to complete.

This survey is being sent to all members of the General Surgeons Australia. Please complete the survey if you routinely treat patients with ASBO as part of your surgical practice. The results of this survey will be used for advocacy purposes, to inform future practice and to determine whether there is surgical equipoise to conduct a randomised clinical trial on the use of nasogastric tubes in the surgical management of ASBO.

By completing this survey, you are indicating your consent to participate in this project. If you are interested in participating in a multisite randomised controlled trial to assess the surgical management of ASBO with or without using a nasogastric tube, you will be given the option to provide your name and email.

Survey responses, once submitted, cannot be reviewed, edited, or withdrawn.

Participation is voluntary; responses will be anonymous. If you do not want to participate, then close this window.

Thank you and kind regards,

A/ Prof Stephen Smith Professor Jonathan Gani

A/ Prof Christine O'Neill Dr Humaira Haider Mahin

John Hunter Hospital, Newcastle.

Demographics

Where is your main work location?	◯ Metropolitan Hospital<br>◯ Regional Hospital<br>◯ Rural Hospital<br>(Select one only.)
What is the postcode of your main work location?	________________________
Have you undertaken subspecialty training/ fellowship?	◯ Yes<br>◯ No

What is your surgical subspecialty/interest?	☐ Breast<br>☐ Endocrine<br>☐ Gastrointestinal<br>☐ Colorectal<br>☐ Trauma<br>☐ Rural General<br>☐ Head and Neck<br>☐ Surgical Oncology<br>☐ Transplant<br>☐ Vascular<br>☐ HPB<br>☐ Bariatric Surgery<br>☐ Other<br>(Select all that apply.)
If other, please specify:	__________________________
How long have you been working as a consultant surgeon?	○ 1-5 years<br>○ 6 -10 years<br>○ 11-15 years<br>○ > 15 years
Do you undertake general surgical / acute surgical on-call?	○ Yes<br>○ No

Adhesional Small Bowel Obstruction Management

How many patients with adhesional small bowel obstruction (ASBO) would you treat per month?	◯ 0-3<br>◯ 4-5<br>◯ 6-10<br>◯ > 10
How frequently would you advise the use of a nasogastric tube (NGT) for the patient with ASBO?	◯ Never<br>◯ 0-10%<br>◯ 11-30%<br>◯ 31-70%<br>◯ 71-90%<br>◯ >90%<br>(Select one only.)
What signs/ symptoms would influence your decision to use an NGT in a patient presenting with ASBO?	☐ Late presentation > 24 hrs since last flatus<br>☐ Abdominal pain<br>☐ Abdominal distension<br>☐ Nausea<br>☐ Vomiting<br>☐ Radiological features of distension<br>☐ Radiological features of threatened small bowel<br>☐ Radiological features of closed-loop small bowel obstruction<br>☐ Patient preference<br>☐ Nursing request<br>☐ Impending need for operative intervention<br>☐ Other reason (please specify)<br>(Select all that apply.)
If other reason, please specify:	__________________________________
What signs/ symptoms would influence your decision NOT to use an NGT in a patient with ASBO?	☐ Early presentation- last flatus < 24hour ago<br>☐ No vomiting<br>☐ No abdominal pain<br>☐ No abdominal distension<br>☐ No focal peritonism<br>☐ Recurrent ASBO<br>☐ Negative inflammatory markers<br>☐ No radiological features of distension<br>☐ Patient preference<br>☐ Nursing request<br>☐ Other reason<br>(Select all that apply.)
If other reason, please specify:	__________________________________
In your opinion, can NGT insertion save some patients with ASBO from operative intervention?	◯ Yes<br>◯ No<br>◯ Undecided/ Not sure
In your opinion, can NGT insertion be therapeutic for ASBO?	◯ Yes<br>◯ No

Inserting a NGT is therapeutic because of?	☐ Preventing the need for surgery<br>☐ Assisting in the resolution of ASBO<br>(Select all that apply.)
Do you think the optimal use of an NGT changes the risk of aspiration in a patient with ASBO?	◯ NGT increases the risk of aspiration<br>◯ NGT does not change the risk of aspiration<br>◯ NGT decreases the risk of aspiration in ASBO<br>◯ Other<br>(Select one only.)
If other, please specify:	__________________________________
What is your preference for the maintenance of an NGT in ASBO?	◯ Free drainage<br>◯ Manual aspiration q4hrly<br>◯ Low continuous suction<br>(Select one only.)
Do you use Gastrograffin during ASBO management?	◯ Yes<br>◯ No
How would you administer Gastrograffin?	◯ Orally<br>◯ Through NGT<br>◯ Either<br>(Select one only.)
Do you insert an NGT before an emergency operation for a patient with ASBO?	◯ No<br>◯ Yes<br>◯ Only if Anaesthetist request<br>◯ Undecided/ Unsure<br>(Select one only.)
How long are you usually prepared to manage ASBO conservatively with an NGT?	◯ 24 hrs<br>◯ 48 hrs<br>◯ 72 hrs<br>◯ 96 hrs<br>◯ Other<br>(Select one only.)
If other, please specify:	____________________________
How long are you usually prepared to manage a patient conservatively when you manage an ASBO patient without a NGT?	◯ 24 hrs<br>◯ 48 hrs<br>◯ 72 hrs<br>◯ 96 hrs<br>◯ 0 hrs<br>(Select one only.)

Clinical Scenarios

A 45-yr old female presented to the emergency department (ED) due to overnight colicky central abdominal pain. Her last bowel action was 24 hrs ago. Laparoscopic appendicectomy was her only significant past medical history. She has not vomitted and has minimal abdominal distension with no tenderness. Her imaging reveals distended proximal small bowel loops with collapsed distal small bowel and no gastric distension.

Would you suggest a NGT for her management?

◯ Yes
◯ No

A 51-yr old male presented to ED with a 1 day history of central abdominal pain. He has vomited several times. His last flatus was 24 hrs ago and he last opened his bowels 48 hrs ago. Two years ago, he had an admission with similar symptoms. Past surgical history includes an open appendicectomy and open bilateral inguinal hernia repair. His CTAP showed multiple dilated small bowel loops with a distended stomach and no distinct transition zone.

Would you suggest a NGT for his management?

◯ Yes
◯ No

A 72-yr old male presented to the ED with a 2-day history of initially intermittent changing to constant generalised abdominal pain, distension and nausea. 30 yrs ago, he had a trauma laparotomy after a motor vehicle accident. His CTAP showed a distended stomach, two adjacent transition zones suggesting a closed-loop mechanism and decreased bowel wall enhancement with diffuse mesenteric haziness.

Would you suggest an NGT as a part of his management?

◯ Yes
◯ No

Survey Comments

Would you consider participating in a randomised controlled trial comparing NGT to no NGT for the management of ASBO?	◯ Yes<br>◯ No<br>(If 'yes' please supply your contact details below.)
Please provide your name:	__________________________________
Please provide an email address:	__________________________________
